# COVID-19-Impfstatus von Pflegenden und assoziierte Faktoren in der stationären Langzeitpflege: Ergebnisse einer Querschnittbefragung im Rahmen des Projekts *Covid-Heim*

**DOI:** 10.1007/s00391-023-02210-2

**Published:** 2023-06-28

**Authors:** Christian Hering, Annabell Gangnus, Raphael Kohl, Elisabeth Steinhagen-Thiessen, Adelheid Kuhlmey, Paul Gellert

**Affiliations:** 1 Institut für Medizinische Soziologie und Rehabilitationswissenschaft, Charité – Universitätsmedizin Berlin, Charitéplatz 1, 10117 Berlin, Deutschland; 2 Medizinische Klinik für Endokrinologie und Stoffwechselmedizin, Charité – Universitätsmedizin Berlin, Augustenburger Platz 1, 13353 Berlin, Deutschland

**Keywords:** COVID-19, Alten- und Pflegeheime, Pflegepersonal, Impfkampagne, COVID-19-Impfung, COVID-19, Nursing homes, Nursing staff, Vaccination campaign, COVID-19 vaccination

## Abstract

**Hintergrund:**

Das Pflegepersonal in der vollstationären Langzeitpflege wurde prioritär geimpft, um die Infektionsgefahr für Bewohnende zu reduzieren und die Arbeitsfähigkeit zu erhalten. Zwar wurde die Impfquote des Pflegepersonals durch eine einrichtungsbezogene Impfpflicht erhöht, Untersuchungen zu assoziierten Faktoren des Impfstatus liegen für Deutschland jedoch nicht vor.

**Ziel:**

Identifikation von assoziierten Faktoren des COVID-19-Impfstatus von Pflegepersonal in der vollstationären Langzeitpflege.

**Methoden:**

Im Zeitraum vom 26.10.2021 bis 31.01.2022 antworteten insgesamt *N* = 1546 Pflegekräfte auf eine Online-Befragung im Rahmen des Projekts Covid-Heim, welche u. a. die Impfkampagne fokussierte und mittels logistischer Regressionsanalysen ausgewertet wurde.

**Ergebnisse:**

Acht von 10 Pflegekräften gaben an, gegen COVID-19 geimpft zu sein (80,6 %). Etwa 7 von 10 Pflegekräften dachten seit Beginn der Pandemie mindestens einige Male daran, ihren Beruf aufzugeben (71,4 %). Ein positiver COVID-19-Impfstatus war assoziiert mit höherem Alter, einer Vollzeitbeschäftigung, COVID-19-Todesfällen in der Einrichtung, einem Tätigkeitsort in Nord- oder Westdeutschland. Häufige Gedanken an eine Aufgabe des Berufs waren mit einem negativen COVID-19-Impfstatus assoziiert.

**Diskussion:**

Die vorliegenden Befunde geben erstmalig Hinweise auf Faktoren, die mit dem COVID-19-Impfstatus von Pflegepersonal in der stationären Langzeitpflege in Deutschland zusammenhängen. Weiterführende quantitative wie qualitative Untersuchungen sind notwendig für ein tieferes Verständnis der Entscheidung für oder gegen eine COVID-19-Impfung bei Pflegenden in der stationären Langzeitpflege, um künftige Impfkampagnen in diesem Bereich zielgerichteter umsetzen zu können.

## Abstract (en)

**Background:**

Nurses working in long-term care facilities were vaccinated first before residents as a matter of priority to protect the latter. Although the vaccination rate of nursing staff eventually rose due to a facility-based vaccination requirement, studies on associated factors of vaccination status are currently not available for the long-term care setting in Germany.

**Objective:**

Associated factors of COVID-19 vaccination status among nursing staff in long-term care facilities were explored.

**Methods:**

An online survey was conducted between October 26th 2021 and January 31st 2022. A total of 1546 nurses working in long-term care in Germany responded to questions concerning the Covid-19 vaccination campaign. Logistic regression analyses were performed.

**Results:**

In this study 8 out of 10 nurses were vaccinated against COVID-19 (80.6%). Approximately 7 out of 10 nurses thought at least a few times about quitting their job since the pandemic began (71.4%). A positive COVID-19 vaccination status was associated with older age, full-time employment, COVID-19 deaths at the facility and working in northern or western Germany. Frequent thoughts of quitting their job were associated with negative COVID-19 vaccination status.

**Conclusion:**

The present findings provide evidence on factors associated with the COVID-19 vaccination status of nurses in long-term care facilities in Germany for the first time. Further quantitative as well as qualitative studies are necessary for a more comprehensive understanding of the COVID-19 vaccination decision-making among nurses in long-term care, in order to implement target-oriented future vaccination campaigns in this care setting.

## Hintergrund

Seit Beginn der COVID-19-Pandemie im März 2020 waren die Einrichtungen der vollstationären Langzeitpflege schwer von deren Folgen betroffen. Eine erhöhte Sterblichkeit und eingeschränkte soziale Teilhabe der Bewohnenden als auch eine erhöhte Arbeitsbelastung der Pflegekräfte konnten beobachtet werden [4, 6, 8, 12]. Daher startete die COVID-19-Impfkampagne priorisiert in den Alten- und Pflegeheimen Deutschlands. Zwar lagen vor Einsetzen der deutschen Impfkampagne keine Zahlen für das dort tätige Pflegepersonal vor, in den USA zeigte sich jedoch eine eingeschränkte Impfbereitschaft von 22–45 % unter den Beschäftigten in der vollstationären Langzeitpflege [19, 21]. Nach Anlauf der Kampagne wurde im Juli 2021 in 14.900 Pflegeheimen in den USA eine durchschnittliche Impfquote von 60 % bei den Beschäftigten festgestellt [14]. Es zeigte sich, dass die Impfquote der Beschäftigten hinter denen der Bewohnenden zurückblieb [2, 20]. Für Deutschland gab es v. a. Erhebungen zur Impfbereitschaft in Krankenhäusern [5, 10, 11, 13]. Im Dezember 2021 wurde die einrichtungsbezogene COVID-19-Impfpflicht im Infektionsschutzgesetz durch Bundestag und Bundesrat beschlossen (§20a IfSG). Die vom „Masernschutzgesetz“ (§20 (8) IfSG) nichtbetroffene stationäre Langzeitpflege sah sich hier erstmals mit einer Impfpflicht konfrontiert. Die anfängliche Wertschätzung für die verrichtete Arbeit der Pflegekräfte in der Pandemie drohte einer wahrgenommenen Geringschätzung zu weichen, da sich Pflegekräfte in ihren individuellen Rechten eingeschränkt sehen konnten [7]. Dies resultierte in einen Diskurs über eine allgemeine Impfpflicht, die jedoch aufgrund fehlender Mehrheiten nicht umgesetzt werden konnte [7]. Im Dezember 2021 erschien der erste Bericht des Robert Koch-Instituts zum Monitoring von COVID-19 und der Impfsituation in Langzeitpflegeeinrichtungen. Es zeigte sich auch hier im Verlauf für das Personal in den Alten- und Pflegeeinrichtungen eine geringere Impfquote (81 %) als bei den Bewohnenden (89 %) oder auch dem Personal im Krankenhaus (92 %) [16, 17]. Befragungen in den USA gaben Hinweise darauf, dass die Trägerschaft, Todesfälle unter den Beschäftigten aufgrund von COVID-19 als auch das Alter der Beschäftigten mit deren Impfbereitschaft assoziiert seien [14, 21]. Aus Deutschland stammende Studien aus dem Krankenhaus- und Wohlfahrtssektor zeigten darüber hinaus Assoziationen zwischen der Akzeptanz des Impfstoffs und dem Kontakt mit COVID-19-infizierten Patient:innen und einem Tätigkeitsort in Westdeutschland [10, 13]. Außerdem zeigte sich, dass einige Pflegekräfte bei Einsetzen einer Impfpflicht eine Kündigung in Erwägung ziehen würden [2, 3, 21]. Dies wurde auch in Deutschland befürchtet und ein Einbruch der Versorgungsleistung prognostiziert und öffentlichkeitswirksam diskutiert [7]. Da es bisher keine Untersuchung gab, die sich mit assoziierten Faktoren mit dem COVID-19-Impfstatus der Pflegekräfte in eben jenem Setting beschäftigt, galt es, diese als Ziel in der vorliegenden Arbeit zu identifizieren.

## Methoden

### Stichprobe

Eine bundesweite Online-Befragung unter Pflegekräften in der vollstationären Langzeitpflege wurde im Zeitraum vom 26.10.2021 bis zum 31.01.2022 durchgeführt. Die Rekrutierung fand in einschlägigen Gruppen auf Facebook wie Altenpflege (76.819 Mitglieder, Stand: 05.01.2023) und über Verteiler des Deutscher Pflegerat e. V. statt. Der Fragebogen wurde über die sichere Webapplikation REDCap bereitgestellt, und sämtliche Daten wurden ausschließlich auf Servern der Charité gespeichert. Für die vorliegenden Analysen lagen Daten von insgesamt 1546 Pflegekräften vor. Die Online-Befragung wurde im Rahmen des vom GKV-Spitzenverband geförderten Projekts *Covid-Heim* durchgeführt, welches auf der Basis unterschiedlicher Datengrundlagen die Folgen der Coronapandemie in der vollstationären Langzeitpflege untersuchte. Ziel des Projekts war es, Lehren für Strukturentwicklungen in diesem Versorgungssetting zu ziehen. Im Ergebnisreport Nr. 6 des Projekts *(Zur COVID-19-Impfkampagne: Ergebnisse aus der zweiten deutschlandweiten Befragung von Heimleitungen und Pflegepersonal)* wurden bereits erste deskriptive Ergebnisse der zugrunde liegenden Befragung sowie aus einer parallel stattgefundenen Online-Befragung von Einrichtungsleitungen veröffentlicht [9]. Aufgrund der hohen gesellschaftlichen Relevanz des Themas sollen mit der vorliegenden Arbeit tiefergehende multivariate Analysen nachgeliefert werden, um Assoziationen zwischen dem COVID-19-Impftstatus der Pflegenden und anderen Faktoren zu identifizieren. Weitere Informationen zum Projekt *Covid-Heim* und der Ergebnisreport Nr. 6 sind auf der Projekthomepage verfügbar unter: https://medizinsoziologie-reha-wissenschaft.charite.de/forschung/alternsforschung/covid_heim/.

### Fragebogen

Um weitestgehend sicherzustellen, dass nur Pflegekräfte aus der stationären Langzeitpflege den Fragebogen beantworten, wurde nach Aufklärung und Einwilligung der derzeitige Tätigkeitsort abgefragt: „Arbeiten Sie derzeit als Pflegekraft in einer vollstationären Alten- und Pflegeeinrichtung (Alten- und Pflegeheim)?“. Nur, wenn „ja“ ausgewählt wurde, konnte der Fragebogen ausgefüllt werden. Bei „nein“ wurde die Befragung abgebrochen.

#### Charakteristika der Stichprobe

Neben Geschlecht, Alter, Anstellungsart, Ausbildung und Tätigkeitsort wurden Größe und Trägerschaft der zugehörigen Einrichtung der Pflegekräfte erfasst. Außerdem wurde erfragt, ob im Laufe der Pandemie eine eigene Infektion mit dem Coronavirus vorlag (Haben Sie sich im Verlauf der Pandemie nachweislich mit dem Coronavirus infiziert? – Ja/nein), ob es seit Beginn der Pandemie (01.03.2020) unter Pflegekräften oder Bewohnenden Todesfälle aufgrund von COVID-19-Infektionen in der Einrichtung gab (Sind seit Beginn der Pandemie [01.03.2020] Bewohner:innen/Kolleg:innen in der Pflege in Ihrer Einrichtung mit oder an einer COVID-19-Infektion verstorben? – Ja/nein), und wie sie die COVID-19-Impfquote der Pflegekräfte als auch der Bewohnenden der Einrichtung in Prozent einschätzen (Wie viel Prozent der Bewohner:innen/Mitarbeiter:innen der Pflege wurden nach Ihrer Einschätzung bereits vollständig gegen das Coronavirus geimpft? Als vollständig geimpft gelten Personen mit mindestens 2facher Impfung der Wirkstoffe von BioNtech, Moderna, AstraZeneca, Kreuzimpfung AstraZeneca und BioNtech/Moderna oder einfache Impfung durch Johnson & Johnson.). Ergänzend dazu wurde der eigene COVID-19-Impfstatus erfragt (Haben Sie sich gegen das Coronavirus impfen lassen? – Ja/nein) und gebeten, darüber Auskunft zu geben, wie häufig es seit Beginn der Pandemie (01.03.2020) Gedanken an eine Aufgabe des Berufs gab (Wie oft haben Sie seit Beginn der Coronapandemie [01.03.2020] daran gedacht, Ihren Beruf aufzugeben? – Nie, einige Male, einige Male im Monat, einige Male in der Woche, jeden Tag) [15]. Darüber hinaus wurden Pflegekräfte, die sich nicht impfen ließen, nach ihrer zukünftigen Impfabsicht gefragt (Können Sie sich vorstellen, sich in Zukunft gegen das Coronavirus impfen zu lassen? – Ja, eher ja, unentschieden, eher nein, nein) [16]. Ebenso wurden Motive erfragt, die die Pflegenden zur Ablehnung oder zur Annahme der Impfung bewegten. Diese wurden bereits im Ergebnisreport Nr. 6 des Projekts COVID-Heim veröffentlicht und sind dort einsehbar [9]. Darüber hinaus enthielt der Fragebogen weitere thematische Schwerpunkte (u. a. körperliche und psychische Gesundheit), die nicht Gegenstand der vorliegenden Arbeit sind.

### Statistische Analysen

Häufigkeiten, Prozentwerte, Mittelwerte und Standardabweichungen wurden für die Charakteristika der Pflegekräfte ausgegeben. Unterschiede zwischen geimpften und ungeimpften Pflegekräften wurden durch Chi-Quadrat-Test und *t*-Test ermittelt. Wo nötig, wurden Post-hoc-Tests durchgeführt. Um assoziierte Faktoren mit dem COVID-19-Impfstatus zu ermitteln, wurde eine logistische Regressionsanalyse durchgeführt. Fälle mit fehlenden Werten wurden aus der Analyse ausgeschlossen (*n* = 394) und mit dem Missing Completely At Random-Test nach Little überprüft (*p* < 0,001). Darüber hinaus wurden eingeschlossene und ausgeschlossene Fälle mit Chi-Quadrat- und *t*-tests verglichen. Berechnungen wurden mit IBM SPSS Statistics für Mac, Version 27.0 (IBM Corp., Armonk, NY, USA) durchgeführt.

## Ergebnisse

### Charakteristika der Stichprobe

An der Online-Befragung haben insgesamt 1546 Pflegekräfte mit einem durchschnittlichen Alter von 39,8 Jahren (± 10,4; *Range* = 16–67) teilgenommen. Der überwiegende Anteil der Befragten war weiblichen Geschlechts (87,6 %). Mehr als die Hälfte absolvierte eine Ausbildung zur Altenpfleger:in (60,3 %). Von den 1546 teilnehmenden Pflegekräften waren insgesamt 80,6 % gegen COVID-19 geimpft. Die Ergebnisse der Chi-Quadrat-Tests und *t*-tests zeigen, dass geimpfte Pflegekräfte älter, häufiger mit COVID-19 infiziert waren, weniger häufig daran dachten, ihren Beruf aufzugeben, eher in Nord- oder Westdeutschland arbeiteten, eher COVID-19-Todesfälle in der Einrichtung erlebten und die Impfquoten von Kolleg:innen und Bewohnenden höher einschätzten im Vergleich zu ungeimpften Pflegekräften. Post-hoc-Tests mit Bonferroni-Korrektur konnten Unterschiede zwischen geimpften und ungeimpften Pflegekräften hinsichtlich Größe und Trägerschaft der Einrichtung nicht bestätigen (Tab. 1).

**Table 1:** 

	*Gesamt* *n (%)*	*Geimpft* *n (valid %)*	*Ungeimpft* *n (valid %)*	*p-Wert*^a,b^
*COVID-19-Impfstaus*				
Geimpft	1246 (80,6)	–	–	–
Ungeimpft	300 (19,4)	–	–	–
*Zukünftige Impfabsicht*				
Ja/eher ja	–	–	35 (11,7)	–
Unentschieden	–	–	62 (20,7)	–
Nein/eher nein	–	–	203 (67,6)	–
*Geschlecht*				0,668^a^
Weiblich	1354 (87,6)	1089 (80,4)	265 (19,6)	–
Männlich	182 (11,8)	148 (81,3)	34 (18,7)	–
Divers	3 (0,2)	3 (100)	0 (0)	–
k. A.	7 (0,5)	–	–	–
*Alter (in Jahren)*				**<** **0,001**^a^
16–29	250 (16,2)	187 (74,8)	63 (25,2)	–
30–39	541 (35,0)	418 (77,3)	123 (22,7)	–
40–49	426 (27,6)	354 (83,1)	72 (16,9)	–
50–59	262 (16,9)	230 (87,8)	32 (12,2)	–
60–67	42 (2,7)	38 (90,5)	4 (9,5)	–
k. A.	25 (1,6)	**–**	–	–
*M* (SD)	39,8 (10,4)	40,4 (10,5)	37,3 (9,6)	**<** **0,001**^b^
*Ausbildung*				0,092^a^
Altenpfleger:in	933 (60,3)	748 (80,2)	185 (19,8)	–
Gesundheits- und Krankenpfleger:in	183 (11,8)	156 (85,2)	27 (14,8)	–
Kranken- bzw. Altenpflegehilfskraft/Pflegeassistent:in	120 (7,8)	87 (72,5)	33 (27,5)	–
Sonstiger Berufsabschluss im Bereich Pflege	122 (7,9)	101 (82,8)	21 (17,2)	–
Kein Berufsabschluss im Bereich Pflege/in Ausbildung	168 (10,9)	136 (81,0)	32 (19,0)	–
k. A.	20 (1,3)	–	–	–
*Anstellungsart*				0,138^a^
Vollzeit	992 (64,2)	810 (81,7)	182 (18,3)	–
Teilzeit	441 (28,5)	347 (78,7)	94 (21,3)	–
Geringfügige Beschäftigung/Sonstige	18 (1,2)	12 (66,7)	6 (33,3)	–
k. A.	95 (6,1)	–	–	–
*COVID-19-Infektion während der Pandemie*				**0,005**^a^
Nein	1221 (79,0)	1001 (82,0)	220 (18,0)	–
Ja	320 (20,7)	240 (75,0)	80 (25,0)	–
k. A.	5 (0,3)	–	–	–
*Gedanken an Aufgabe des Berufs seit Beginn der Pandemie*^c^				**<** **0,001**^a^
Nie	246 (15,9)	**217 (88,2)**	**29 (11,8)**	**<** **0,001**^d^
Einige Male	366 (23,7)	300 (82,0)	66 (18,0)	0,368^d^
Einige Male im Monat	234 (15,1)	194 (82,9)	40 (17,1)	0,271^d^
Einige Male in der Woche	251 (16,2)	194 (77,3)	57 (22,7)	0,193^d^
Jeden Tag	254 (16,4)	**180 (70,9)**	**74 (29,1)**	**<** **0,001**^d^
k. A.	195 (12,6)	–	–	–
*Tätigkeitsort*				**<** **0,001**^a^
Ostdeutschland^e^	351 (22,7)	**248 (70,7)**	**103 (29,3)**	**<** **0,001**^f^
Süddeutschland^g^	385 (24,9)	295 (76,6)	90 (23,4)	0,016^f^
Norddeutschland^h^	302 (19,5)	**263 (87,1)**	**39 (12,9)**	**0,002**^f^
Westdeutschland^i^	485 (31,4)	**425 (87,6)**	**60 (12,4)**	**<** **0,001**^f^
k. A.	23 (1,5)	–	–	–
*Trägerschaft der Einrichtung*				**0,047**^a^
Privat	659 (42,6)	518 (78,6)	141 (21,4)	0,089^j^
Öffentlich	542 (35,1)	435 (80,3)	107 (19,7)	0,764^j^
Freigemeinnützig	336 (21,7)	**286 (85,1)**	**50 (14,9)**	0,016^j^
k. A.	9 (0,6)	–	–	–
*Größe der Einrichtung*				**0,045**^a^
Klein (1–50 Bewohnende)	298 (19,3)	225 (75,5)	73 (24,5)	0,020^j^
Mittel (51–100 Bewohnende)	756 (48,9)	615 (81,3)	141 (18,7)	0,420^j^
Groß (≥ 101 Bewohnende)	483 (31,2)	398 (82,4)	85 (17,6)	0,230^j^
k. A.	9 (0,6)	–	–	–
*COVID-19-Todesfälle in der Einrichtung*^k^				**0,045**^a^
Nein	623 (40,3)	487 (78,2)	136 (21,8)	–
Ja	915 (59,2)	753 (82,3)	162 (17,7)	–
k. A.	8 (0,5)	–	–	–
*Schätzung der Impfquote der Bewohnenden in der Einrichtung (in %)*				
*n* (%)	1503 (97,2)	1213 (80,7)	290 (19,3)	–
M (SD)	87,9 (12,6)	88,6 (11,3)	85,1 (16,6)	**<** **0,001**^b^
*Schätzung der Impfquote der Kolleg:innen in der Einrichtung (in %)*				
*n* (%)	1493 (96,6)	1208 (80,9)	285 (19,1)	–
M (SD)	77,6 (17,3)	79,5 (16,0)	69,7 (20,4)	**<** **0,001**^b^

Statistisch signifikante Werte sind fett gedruckt

*M* Mittelwert, *SD* Standardabweichung

^a^Pearson-Chi-Quadrat Test

^b^*t*-Test für unabhängige Stichproben

^c^Als Beginn der Pandemie wurde hier der 01.03.2020 definiert

^d^Post-hoc-Test mit Bonferroni-Korrektur; sign. *p* < 0,005

^e^Brandenburg, Berlin, Thüringen, Sachsen, Sachsen-Anhalt

^f^Post-hoc-Test mit Bonferroni-Korrektur; sign. *p* < 0,006

^g^Bayern, Baden-Württemberg

^h^Mecklenburg-Vorpommern, Niedersachsen, Schleswig-Holstein, Bremen, Hamburg

^i^Nordrhein-Westfalen, Rheinland-Pfalz, Saarland, Hessen

^j^Post-hoc-Test mit Bonferroni-Korrektur; sign. *p* < 0,008

^k^Todesfälle unter Bewohnenden und/oder Kolleg:innen

### Logistische Regressionsanalyse

Insgesamt gingen in die logistische Regressionsanalyse *n* = 1152 Fälle ein. Eingeschlossene Fälle waren, verglichen mit ausgeschlossenen Fällen, eher weiblichen Geschlechts (89,4 % vs. 84,4 %; *p* < 0,01), älter (40,3 Jahre vs. 38,1 Jahre; *p* < 0,001) und gaben eine höhere Schätzung der Impfquote der Bewohner:innen an (88,5 % vs. 86,1 %; *p* < 0,01). Die Ergebnisse zeigen signifikante Assoziationen zwischen dem Impfstatus der Pflegekräfte und dem Alter (OR 1,03, 95 %-KI 1,01–1,05), einer Vollzeitbeschäftigung (OR 1,56, 95 %-KI 1,10–2,22), COVID-19-Todesfällen in der Einrichtung (OR 1,46, 95 %-KI 1,02–2,10) als auch der Schätzung der Impfquote der Pflegekräfte in der Einrichtung (OR 1,01, 95 %-KI 1,02–1,04). Darüber hinaus besteht eine Assoziation zwischen dem Tätigkeitsort der Pflegekräfte und dem COVID-19-Impfstatus. Im Vergleich zu Ostdeutschland geben Pflegekräfte in Nord- und Westdeutschland signifikant häufiger an, gegen COVID-19 geimpft zu sein (OR 1,23, 95 %-KI 1,23–3,52; OR 2,26, 95 %-KI 1,42–3,60). Außerdem sind Pflegekräfte, die einige Male in der Woche oder jeden Tag seit Beginn der Pandemie an die Aufgabe ihres Berufs gedacht haben, signifikant häufiger nicht gegen COVID-19 geimpft im Vergleich zu Pflegekräften, die nie darüber nachgedacht haben (OR 0,54, 95 %-KI 0,31–0,95; OR 0,36, 95 %-KI 0,21–0,62; Tab. 2).

**Table 2:** 

	COVID-19-Impftstatus: geimpft		
	*OR*	*95* *%-KI*	*p‑Wert*
Geschlecht (weiblich)	0,98	0,56–1,69	0,929
Alter (in Jahren)	**1,03**	**1,01–1,05**	**0,001**
*Ausbildung*			
Altenpfleger:in [Referenz]	–	1	–
Gesundheits- und Krankenpfleger:in	1,78	0,98–3,22	0,057
Kranken- bzw. Altenpflegehilfskraft/Pflegeassistent:in	0,74	0,42–1,30	0,287
Sonstiger Berufsabschluss im Bereich Pflege	1,44	0,77–2,71	0,256
Kein Berufsabschluss im Bereich Pflege/in Ausbildung	0,91	0,54–1,55	0,726
Anstellungsart (Vollzeit)	**1,56**	**1,10–2,22**	**0,013**
COVID-19-Infektion während der Pandemie (ja)	0,77	0,52–1,13	0,186
*Gedanken an Aufgabe des Berufs seit Beginn der Pandemie*^*a*^			
Nie [Referenz]	–	1	–
Einige Male	0,77	0,45–1,32	0,341
Einige Male im Monat	0,77	0,43–1,37	0,366
Einige Male in der Woche	**0,54**	**0,31–0,95**	**0,033**
Jeden Tag	**0,36**	**0,21–0,62**	**<** **0,001**
*Tätigkeitsort*			
Ostdeutschland^b^ [Referenz]	–	1	–
Süddeutschland^c^	1,10	0,71–1,66	0,706
Norddeutschland^d^	**2,10**	**1,23–3,52**	**0,006**
Westdeutschland^e^	**2,26**	**1,42–3,60**	**0,001**
*Trägerschaft der Einrichtung*			
Privat [Referenz]	–	1	–
Öffentlich	1,12	0,77–1,61	0,557
Freigemeinnützig	1,48	0,94–2,34	0,090
*Größe der Einrichtung*			
Klein (1–50 Bewohnende) [Referenz]	–	1	–
Mittel (51–100 Bewohnende)	1,12	0,76–1,77	0,505
Groß (≥ 101 Bewohnende)	1,11	0,70–1,78	0,652
COVID-19-Todesfälle in der Einrichtung^f^ (ja)	**1,46**	**1,02–2,10**	**0,042**
Schätzung der Impfquote der Bewohnenden der Einrichtung (in %)	1,00	0,98–1,01	0,504
Schätzung der Impfquote der Kolleg:innen in der Einrichtung (in %)	**1,01**	**1,02–1,04**	**<** **0,001**

Statistisch signifikante Werte sind fett gedruckt

*OR* Odds Ratio, *KI* Konfidenzintervall

^a^Als Beginn der Pandemie wurde hier der 01.03.2020 definiert

^b^Brandenburg, Berlin, Thüringen, Sachsen, Sachsen-Anhalt

^c^Bayern, Baden-Württemberg

^d^Mecklenburg-Vorpommern, Niedersachsen, Schleswig-Holstein, Bremen, Hamburg

^e^Nordrhein-Westfalen, Rheinland-Pfalz, Saarland, Hessen

^f^Todesfälle unter Bewohnenden und/oder Kolleg:innen

## Diskussion

Die Ergebnisse der vorliegenden Untersuchung konnten Assoziationen zwischen dem COVID-19-Impfstatus des Pflegepersonals und höherem Alter, einer Vollzeitbeschäftigung, einem Tätigkeitsort in West- oder Norddeutschland, einige Male in der Woche oder jeden Tag seit Beginn der Pandemie daran zu denken, den Beruf aufzugeben, COVID-19-Todesfällen in der Einrichtung sowie einer höheren Einschätzung der Impfquote der Kolleg:innen in der Einrichtung identifizieren.

Die in der vorliegenden Arbeit ermittelte Impfquote kommt den vom Robert Koch-Institut für denselben Zeitraum und dasselbe Setting ausgewiesenen Impfquoten nah (81–89 %). Zwar stieg nach Beschluss der einrichtungsbezogenen Impfpflicht im Dezember 2021 die Impfquote der Mitarbeitenden in der vollstationären Langzeitpflege [17], jedoch wurde eine Abwanderung von Pflegekräften – bei bestehendem und seit Jahren fortschreitendem Mangel – durch deren Einführung befürchtet [2, 3, 7, 21]. Die Assoziation zwischen negativen COVID-19-Impftstatus und häufigen Gedanken an eine Aufgabe des Berufs könnte implizieren, dass im Zusammenhang mit der Umsetzung der einrichtungsbezogenen Impfpflicht und der ohnehin hohen Belastung in der Pandemie erneut Pflegekräfte den Beruf verlassen haben könnten. Darüber hinaus bestätigten unsere Ergebnisse frühere Untersuchungen in den USA, die nahelegten, dass die Annahme des COVID-19-Impfstoffs mit zunehmendem Alter und Todesfällen in der Einrichtung assoziiert ist [16, 21]. Es zeigte sich außerdem, dass Pflegekräfte in Nord- und Westdeutschland signifikant häufiger gegen COVID-19 geimpft waren, im Vergleich zu deren Kollegen in der Referenzkategorie Ostdeutschland, von der sich Süddeutschland wiederum nicht signifikant unterschied. Dies unterstützt Befunde, die zeigen, dass in Deutschland, je nach Region, Unterschiede bei der Impfbereitschaft von Gesundheitspersonal zu existieren scheinen [11, 14]. Eine höhere Einschätzung der Impfquote unter den Kolleg:innen schien ebenfalls mit der Entscheidung für eine COVID-19-Impfung assoziiert zu sein. So beschrieben Dennis et al. [2] bereits den sozialen Druck als Einflussfaktor auf die Impfbereitschaft von Pflegeheimpersonal in England. Es ist jedoch zu erwähnen, dass die in der vorliegenden Arbeit gefundenen signifikanten Assoziationen zwischen zunehmendem Alter sowie Schätzung der Impfquote der Kolleg:innen und positivem COVID-19-Impfstatus als gering und damit in ihrer Relevanz zu relativieren sind. Zugrunde liegende Motive für die Ablehnung oder Annahme der COVID-19-Impfung wurden ebenso erhoben und im Ergebnisreport Nr. 6 des Projekts *COVID-Heim* veröffentlicht [9]. Vor allem die Skepsis gegenüber der Wirksamkeit der zugelassenen Impfstoffe, Ängste/Befürchtungen vor Langzeitfolgen und Nebenwirkungen sowie eine Skepsis gegenüber der wissenschaftlichen Fundierung spielten hier die größte Rolle, sich gegen eine COVID-19-Impfung zu entscheiden. Ähnliche Motive wurden auch für Mitarbeitende in Pflegeheimen in Belgien und England beschrieben [2, 3]. Zukünftige Untersuchungen sollten den Impfstatus in der vollstationären Pflege auch vor dem Hintergrund des „5C-Modells“ untersuchen, um die Entscheidung für oder gegen eine Impfung auch in diesem Setting auch psychologisch noch besser nachvollziehen zu können. Hier werden Vertrauen („confidence“), Risikowahrnehmung („complacency“), Barrieren („constraints“), Engagement bei der Informationssuche („calculation“) und kollektive Verantwortung („collective responsibility“) als maßgeblich beteiligte Faktoren bei der Impfentscheidung postuliert [1].

### Stärken und Limitationen

Zu den Stärken der vorliegenden Studie zählt die für diese Berufsgruppe große Stichprobe. Außerdem ist die vorliegende Untersuchung nach unserem Kenntnisstand die erste, die sich mit assoziierten Faktoren des COVID-19-Impftstaus von Pflegepersonal in der vollstationären Langzeitpflege beschäftigt. Zu den Limitationen zählt, dass durch die Online-Befragung Pflegekräfte, die über einen eingeschränkten Internetzugang verfügen oder keine Konten in sozialen Medien besitzen, unterrepräsentiert sind. Zeitliche Ressourcen, ein allgemeines Interesse am Thema und die Teilnahmebereitschaft des Pflegepersonals können ebenso zu einem Selektionsbias beitragen. Außerdem zeigten sich Pflegekräfte im Vergleich zur Pflegestatistik jünger und überdurchschnittlich gut ausgebildet in unserer Stichprobe [18]. Darüber hinaus müssen die Ergebnisse vor dem Hintergrund der Assoziation von Geschlecht und Alter auf fehlende Werte in der Analyse betrachtet werden. Zwar ist somit von einer Einschränkung der Generalisierbarkeit auszugehen, die Ergebnisse liefern dennoch erste Anhaltspunkte für eine Verbesserung zukünftiger Impfkampagnen.

## Fazit für die Praxis

Eine Impfkampagne, die Faktoren wie Alter, Art der Beschäftigung, Tätigkeitsort und Betroffenheit durch COVID-19 oder eine andere Infektionskrankheit miteinbezieht und stärker auf umfassende und transparente Aufklärung der professionell Pflegenden setzt, könnte in Zukunft dazu beitragen, mehr Pflegekräfte für eine freiwillige Annahme einer Impfung zu gewinnen. Vor allem die Transparenz hinsichtlich der wissenschaftlichen Fundierung, Wirkweise und möglicher Nebenwirkungen sollten erläutert und Ängste und Bedenken der Pflegekräfte ernst genommen und adressiert werden.

## References

[1] Betsch C, Schmid P, Korn L (2019). Impfverhalten psychologisch erklären, messen und verändern. Bundesgesundheitsblatt Gesundheitsforschung Gesundheitsschutz.

[2] Dennis A, Robin C, Jones LF (2022). Exploring vaccine hesitancy in care home employees in North West England: a qualitative study. BMJ Open.

[3] Digregorio M, Van Ngoc P, Delogne S (2022). Vaccine hesitancy towards the COVID-19 vaccine in a random national sample of belgian nursing home staff members. Vaccines.

[4] Gangnus A, Hering C, Kohl R (2021). Covid-19-Schutzmaßnahmen und Einschränkungen des sozialen Lebens in Pflegeheimen: Analyse von Verordnungen und Surveydaten. Pflege.

[5] Ganslmeier A, Engelmann T, Lucke M (2021). Einstellung von Pflegekräften zur SARS-CoV-2-Impfung. MMW Fortschr Med.

[6] Gardner W, States D, Bagley N (2020). The Coronavirus and the risks to the elderly in long-term care. J Aging Soc Policy.

[7] Gräske J, Forbrig TA (2022). Mögliche Folgen der Einrichtungsbezogenen Impfpflicht nach § 20a Infektionsschutzverordnung – eine Querschnittserhebung von Einrichtungen nach SGB V und SGB XI.

[8] Hering C, Gangnus A, Budnick A (2022). Psychosocial burden and associated factors among nurses in care homes during the COVID-19 pandemic: findings from a retrospective survey in Germany. BMC Nurs.

[9] Hering C, Gangnus A, Kohl R et al (2022) Lehren aus der Corona-Pandemie für Strukturentwicklungen im Versorgungssetting Pflegeheim. Ergebnisreport Nr. 6. Zur Covid-19-Impfkampagne: Ergebnisse aus der zweiten deutschlandweiten Befragung von Heimleitungen und Pflegepersonal. https://medizinsoziologie-reha-wissenschaft.charite.de/fileadmin/user_upload/microsites/m_cc01/medizinsoziologie-reha-wissenschaft/Dateien_Forschung/Alternsforschung/CovidHeim_ErgebnisReport6_31_01_2022.pdf. Zugegriffen: 15.11.2022

[10] Holzmann-Littig C, Braunisch MC, Kranke P (2021). COVID-19 vaccination acceptance and hesitancy among healthcare workers in Germany. Vaccines.

[11] Janssens U, Kluge S, Marx G (2021). Einstellung zur Impfung gegen SARS-CoV-2. Med Klin Notfallmed.

[12] Kohl R, Schwinger A, Jürchott K (2022). Mortality among hospitalized nursing home residents with COVID-19. Dtsch Ärztebl Int.

[13] Kozak A, Nienhaus A (2021). COVID-19 vaccination: status and willingness to be vaccinated among employees in health and welfare care in Germany. Int J Environ Res Public Health.

[14] McGarry BE, Shen K, Barnett ML (2021). Association of nursing home characteristics with staff and resident COVID-19 vaccination coverage. JAMA Intern Med.

[15] Nübling M, Stößel U, Hasselhorn H‑M (2006). Measuring psychological stress and strain at work-Evaluation of the COPSOQ Questionnaire in Germany. GMS. Psychosoc Med.

[16] Robert Koch-Institut (2021) KROCO – die Krankenhausbasierte Online-Befragung zur COVID-19-Impfung. Ergebnisbericht zur Dritten Befragungswelle. https://www.rki.de/DE/Content/InfAZ/N/Neuartiges_Coronavirus/Projekte_RKI/Kroco-Report100122.pdf?__blob=publicationFile. Zugegriffen: 15.11.2022

[17] Robert Koch-Institut (2022) Monitoring von COVID-19 und der Impfsituation in Langzeitpflegeeinrichtungen – Stand der Erhebung September bis November 2021. 2. Bericht vom 13.01.2022. https://www.rki.de/DE/Content/Infekt/Impfen/ImpfungenAZ/COVID-19/Bericht2_Monitoring_COVID-19_Langzeitpflegeeinrichtungen.pdf?__blob=publicationFile. Zugegriffen: 15.11.2022

[18] Statistisches Bundesamt (2020) Pflegestatistik 2019. Wiesbaden 2019. https://www.destatis.de/DE/Themen/Gesellschaft-Umwelt/Gesundheit/Pflege/Publikationen/Downloads-Pflege/pflege-deutschlandergebnisse-5224001199004.pdf?__blob=publicationFile. Zugegriffen: 15.11.2022

[19] Susheela A (2021). COVID-19 vaccine hesitancy in nursing: Home staff and the need for ongoing education and vaccine access. Am J Aging Sci Res.

[20] Tulloch JS, Lawrenson K, Gordon AL (2023). COVID-19 vaccine hesitancy in care home staff: a survey of Liverpool care homes. Vaccine.

[21] Unroe KT, Evans R, Weaver L (2021). Willingness of long-term care staff to receive a COVID-19 vaccine: a single state survey. J Am Geriatr Soc.

